# Verrucomicrobial community structure and abundance as indicators for changes in chemical factors linked to soil fertility

**DOI:** 10.1007/s10482-015-0530-3

**Published:** 2015-07-17

**Authors:** Acacio Aparecido Navarrete, Tielle Soares, Raffaella Rossetto, Johannes Antonie van Veen, Siu Mui Tsai, Eiko Eurya Kuramae

**Affiliations:** Cell and Molecular Biology Laboratory, Center for Nuclear Energy in Agriculture CENA, University of São Paulo USP, Piracicaba, SP Brazil; Agency for Agribusiness Technology APTA, Piracicaba, SP Brazil; Department of Microbial Ecology, Netherlands Institute of Ecology NIOO-KNAW, Droevendaalsesteeg 10, 6708 PB Wageningen, The Netherlands

**Keywords:** Bioindicators, Tropical soils, Slash-and-burn, Land-use changes, Sugarcane

## Abstract

Here we show that verrucomicrobial community structure and abundance are extremely sensitive to changes in chemical factors linked to soil fertility. Terminal restriction fragment length polymorphism fingerprint and real-time quantitative PCR assay were used to analyze changes in verrucomicrobial communities associated with contrasting soil nutrient conditions in tropical regions. In case study Model I (“Slash-and-burn deforestation”) the verrucomicrobial community structures revealed disparate patterns in nutrient-enriched soils after slash-and-burn deforestation and natural nutrient-poor soils under an adjacent primary forest in the Amazonia (*R* = 0.819, *P* = 0.002). The relative proportion of *Verrucomicrobia* declined in response to increased soil fertility after slash-and-burn deforestation, accounting on average, for 4 and 2 % of the total bacterial signal, in natural nutrient-poor forest soils and nutrient-enriched deforested soils, respectively. In case study Model II (“Management practices for sugarcane”) disparate patterns were revealed in sugarcane rhizosphere sampled on optimal and deficient soil fertility for sugarcane (*R* = 0.786, *P* = 0.002). Verrucomicrobial community abundance in sugarcane rhizosphere was negatively correlated with soil fertility, accounting for 2 and 5 % of the total bacterial signal, under optimal and deficient soil fertility conditions for sugarcane, respectively. In nutrient-enriched soils, verrucomicrobial community structures were related to soil factors linked to soil fertility, such as total nitrogen, phosphorus, potassium and sum of bases, i.e., the sum of calcium, magnesium and potassium contents. We conclude that community structure and abundance represent important ecological aspects in soil verrucomicrobial communities for tracking the changes in chemical factors linked to soil fertility under tropical environmental conditions.

**Electronic supplementary material:**

The online version of this article (doi:10.1007/s10482-015-0530-3) contains supplementary material, which is available to authorized users.

## Introduction

Members of the phylum *Verrucomicrobia* have been shown to make up 1–10 % of the total bacterial 16S rRNA in soils (Buckley and Schmidt 2001, 2003; Lupatini et al. 2013; Navarrete et al. 2015a, b). Bergmann et al. (2011) recognized the dominance of *Verrucomicrobia* in soil bacterial communities across a range of biomes in Antarctica, Europe, and the Americas. The broad distribution of *Verrucomicrobia* in soils suggests that they are important members of terrestrial ecosystems (Buckley and Schmidt 2001; Felske and Akkermans 1998). Despite the fact that members of this phylum have typically been recalcitrant to cultivation methods (Janssen et al. 1997, 2002; Janssen 1998; Joseph et al. 2003; Davis et al. 2005), new strategies for isolation, as well as novel methods for detection of sought-after microorganisms on solid media, have yielded more *Verrucomicrobia* isolates (Stevenson et al. 2004; Sangwan et al. 2005; Pol et al. 2007; Islam et al. 2008). As a consequence, genomic and physiological characterizations of *Verrucomicrobia* isolates have contributed to the knowledge of their biology and ecology (Isanapong et al. 2012; Wertz et al. 2012). However, most information on the ecology of *Verrucomicrobia* is revealed by the correlations of 16S rRNA gene abundance with environmental parameters, such as soil moisture and soil physicochemical factors (Buckley and Schmidt 2001; Jordaan and Bezuidenhout 2013; Pan et al. 2014).

The culture-independent approach based on the direct recovery of bacterial 16S rRNA genes from tropical soils has revealed the occurrence of *Verrucomicrobia* from different Brazilian biomes: Amazon (Borneman and Triplett 1997; Kim et al. 2007; Navarrete et al. 2010, 2015b), Atlantic forest (Bruce et al. 2010), Cerrado (Quirino et al. 2009) and Pampa (Lupatini et al. 2013). However, the information acquired is still not sufficient as a systematic identification of taxa responding to the alterations in soil chemical factors. In soils from the Amazon region, *Verrucomicrobia* were present at different abundance under diverse soil management practices, opening the possibilities to investigate verrucomicrobial community as bioindicator of tropical soil management effects.

Biological processes, species or communities can serve as successful bioindicators (Holt and Miller 2011). A new perspective in microbial ecology has emerged due to the progresses of molecular biology, allowing the interpretation of ecological aspects by culture-independent approaches. Microbial community structure, including the number of species and the relative abundance of species, has been assessed by molecular fingerprinting techniques (Burlage 1998). Microbial community abundance, an ecological concept referring to the relative representation of a community in a particular ecosystem, can be estimated using quantitative molecular approaches (Smith and Osborn 2009). These ecological aspects of microbial communities can be statistically related to environmental parameters, such as soil chemical factors (Jesus et al. 2009; Kuramae et al. 2010, 2012; Navarrete et al. 2013; Pan et al. 2014).

In the present study, we examined changes in the verrucomicrobial community associated with high soil fertility after slash-and-burning deforestation in the Amazonia (Model I), and decline in soil fertility associated with different management practices for sugarcane (Model II). For this purpose, the verrucomicrobial community structure was assessed by terminal restriction fragment polymorphism (T-RFLP) analysis, and the verrucomicrobial community abundance was estimated by real-time quantitative PCR (qPCR). The explicit relationship between the verrucomicrobial community structure and soil chemical factors was examined by multivariate statistical analyses.

## Materials and methods

Soil was sampled in two contrasting soil nutrient content situations: Model I (Slash-and-burn deforestation): soil samples were collected in nutrient-enriched soils after slash-and-burn deforestation and natural nutrient-poor soils under adjacent primary forest in three discontinuous areas in the Amazonian region. Model II (Management practices for sugarcane): soil samples were collected from sugarcane rhizosphere with optimal and deficient soil nutrients in a greenhouse mesocosm experiment. Fieldwork was conducted under legal authorization (SISBIO 4845833).

### Model I: slash-and-burn deforestation

#### Study sites and soil sampling

Soil samples were collected in three discontinuous areas located in the Southeastern Brazilian Amazon, State of Mato Grosso, Brazil: area 1 (15°11′45″S and 59°03′31″W), area 2 (14°21′38″S and 57°21′27″W) and area 3 (13°21′57″S and 54°54′24″W) described previously (Navarrete et al. 2015b). The three discontinuous sampling areas were considered replicates, and soil samples were collected from deforested sites identified just after forest clearing and adjacent primary forest sites exactly as described in Navarrete et al. (2015b). Samples were transported to the laboratory under ice and stored at −20 °C until processing within 72 h after sampling. Soil fertility properties were determined previously for the same soil samples used in this study by Navarrete et al. (2015a, b) (Supplementary Table 1).

#### Soil DNA isolation and verrucomicrobial 16S rDNA T-RFLP fingerprinting analysis

DNA was extracted from 250 mg soil samples from deforested and forest sites using the Power Soil DNA Isolation Kit (Mo Bio Laboratories Inc., Carlsbad, CA, USA), according to the manufacturer’s instructions. DNA extraction was performed in duplicate for each soil sample and quantified using Qubit dsDNA BR Assay Kit (Invitrogen, Carlsbad, CA, USA). DNA concentrations were adjusted to 20 ng µl^−1^ and stored at −20 °C until use. T-RFLP analysis was used to characterize the verrucomicrobial community structure in soils from deforested sites and forest sites. Verrucomicrobial 16S rRNA gene fragments were amplified in duplicate 25 μl reactions using the primers VMB537f (O’Farrell and Janssen 1999) and 1378r (Heuer et al. 1997). The forward primer was labeled with hexacarboxifluorescein at the 5′end. Each 25 μl reaction mixture contained 2.5 μl of reaction buffer 10× (Invitrogen, Carslad, CA, USA), 0.75 μl of MgCl_2_ (50 mM), 0.5 μl of each primer (10 µM), 0.2 U of Platinum *Taq* DNA Polymerase (Invitrogen), 0.5 μl of each dNTP (1 mM), 10 ng of bovine serum albumin (BSA; 10 mg ml^−1^) and 10 ng of template DNA. The following conditions were chosen for amplification after optimization: initial denaturation for 5 min at 95 °C; 35 cycles of 1 min at 95 °C, 30 s at 60 °C, and 1.5 min at 72 °C; and final extension for 10 min at 72 °C. The duplicate hexacarboxifluorescein-labeled PCR products for each sample were pooled and purified using GFX™ PCR DNA and Gel Band Purification Kit (Amersham Pharmacia Biotech, NJ, USA) after analysis by gel electrophoresis. Purified products were split into three tubes (175 ng in each tube) and digested in separate 15 μl-reactions with 10 U of the restriction enzymes *Alu*I, *Msp*I and *Hha*I (Invitrogen, Carlsbad, CA, USA) for 3 h at 37 °C. Fluorescently labeled terminal restriction fragments (TRF) were separated and detected using an ABI PRISM 3100 Genetic Analyzer capillary sequencer (Applied Biosystems, Foster City, CA, USA). Before injection, the samples were precipitated using sodium acetate/EDTA, and denatured in the presence of 10 μl formamide and 0.25 μl GS-500 ROX size standard (Applied Biosystems, Foster City, CA, USA). The TRF patterns were evaluated using Peak Scanner (Applied Biosystems) and T-REX (http://trex.biohpc.org/) software. TRFs were defined by aligning peaks using clustering threshold (range specified equal to 1.0). TRFs <50 bp or contributing to 0.5 % of the total TRF signal were excluded. Matrices (concatenated matrix with the three enzymes) for presence/absence were analyzed using CANOCO 4.5 (ter Braak and Šmilauer 2002) to generate ordination of T-RFLP patterns by principal component analysis (PCA). A distance matrix (Jaccard metric) was constructed from presence/absence data. This similarity matrix was used for ANOSIM statistics to investigate differences in soil verrucomicrobial community structure inhabiting nutrient-enriched soils after slash-and-burn deforestation in the Amazonia and natural nutrient-poor soils under adjacent primary forest. The magnitude of *R* indicates the degree of separation between two communities, with a score of ‘1’ indicating complete separation and ‘0’ indicating no separation. Calculation of similarity coefficient and ANOSIM were carried out using Primer six (version 6.1.5, Primer-E Ltd., Plymouth, UK). The explicit relationship between the verrucomicrobial community structure and soil chemical factors was examined by constrained ordination generated by a redundancy analysis (RDA) performed using CANOCO 4.5 (ter Braak and Šmilauer 2002).

#### Quantitative real-time PCR assay

Quantitative real-time PCR (qPCR) using the 16S rRNA marker gene was performed to assess the abundance of the verrucomicrobial and total bacterial communities using the same soil DNA samples as those for T-RFLP fingerprinting. As standards, 16S rRNA gene amplicons from *Verrucomicrobia spinosum* (DSMZ 4136) and a bacterial clone from an environmental sample were obtained by PCR using primers pA-F (5′-AGAGTTTGATCCTGGCTCAG-3′) (Edwards et al. 1989) and 1378R (5′-CGGTGTGTACAAGGCCCGGGAAGG-3′) (Heuer et al. 1997), purified (QIA-quick PCR purification kit, Qiagen, Venlo, the Netherlands) and ligated into the pGEM-T vector (Promega, Leiden, the Netherlands). Ligation products were transformed with *E. coli* JM109 competent cells (Promega, Leiden, the Netherlands). The presence or absence of the insert was determined by PCR using SP6 and T7 primers, and plasmid DNA was isolated (QIAprep Spin Miniprep Kit, Qiagen, Venlo, the Netherlands) from appropriate clones (i.e., belonging to the desired target group). DNA standard curves were generated by producing a dilution series of 10^4^–10^8^ copies µl^−1^ using duplicate ten-fold dilutions of isolated plasmid DNA. For qPCR of 16S rRNA gene fragments from *Verrucomicrobia* and from total bacteria, we used the following primer pairs: Ver53/Eub518 and Eub338/Eub518, respectively (Lane 1991; Muyzer et al. 1993; Stevenson et al. 2004). Each 25 µl reaction contained 12.5 µl QPCR SYBR green 2× reaction mix (Abgene, Epsom, UK), 1.25 µl of each primer (30 µM), 2.5 µl of bovine serum albumin (BSA; 10 mg ml^−1^) and 50 ng of template DNA. All mixes were made using a CAS-1200 pipetting robot (Corbett Research, Sydney, Australia). PCR conditions for *Verrucomicrobia* and total bacteria were described by Fierer et al. (2005) with the modification of annealing temperature (60 °C) and forward primer (Ver53) in the case of *Verrucomicrobia*. PCR amplifications and product quantification were performed using the Rotor-Gene 3000 (Corbett Research, Sydney, Australia). Melting curve analysis of amplicons was conducted to confirm that fluorescent signals originated from specific amplicons and not from primer-dimers or other artifacts. DNA samples were processed using five replicate reactions for each of six sampling sites in field. Automated analysis of PCR amplicon quality (for example, PCR baseline subtraction, Ct-threshold setting to the linear amplification phase) and quantity was performed with ROTOR-GENE 6 software (Corbett Research, Sydney, Australia). Statistical analyses of qPCR data were performed using the STATISTICA 10 package (StatSoft Inc, Tulsa, OK, USA). One-way ANOVA was used to determine significance of the differences among all soil samples. The comparison of soil samples was based on post hoc analysis using Tukey’s HSD test.

### Model II: management practices for sugarcane

#### Experimental design, treatments and soil sampling

Sugarcane plants (*Saccharum* spp.) were grown in a greenhouse mesocosm experiment as described previously (Navarrete et al. 2015a). Six treatments and three replications were used in a completely randomized design. Mineral fertilizer was applied in the form of urea (450 g N kg^−1^) at a rate of 60 kg N ha^−1^ in treatments containing nitrogen fertilizer. Vinasse, a liquid residue of the ethanol distillation, was applied to the soil at a rate of 0.06 L kg^−1^ (120 m^3^ ha^−1^) in treatments containing vinasse as fertilizer. The experiment consisted of two conditions of soil-surface straw coverage: dry and chopped leaves from adult sugarcane plants straw (10 t ha^−1^) and uncovered surface. Accordingly, the experiment included the following treatments: N, nitrogen fertilizer; N + S, N fertilizer and straw; N + V, N and vinasse as fertilizers; N + V + S, N and V as fertilizers and straw; C, excluding any N, V fertilizer and straw (control); and C + S, excluding any N and V fertilizer and including straw. Ten sugarcane plants were grown in each mesocosm. Sugarcane plants were removed in pairs from each mesocosm at 50 and 150 days after planting and soil fertilization on the optimal and deficient soil fertility conditions for sugarcane, respectively, according to van Raij et al. (1996). Plants were healthy at 50 days after planting and they presented nutrient deficiency symptoms at 150 days after planting. Roots and associated soil were transported to the laboratory on ice and then processed to obtain the rhizosphere soil. The roots were shaken to remove the loose soil, and the tightly attached soil including small aggregates (< 0.5 cm) was used for DNA extraction. The fertility status of the soil on optimal and deficient soil nutrients in each experimental treatment was determined previously by Navarrete et al. (2015a) (Supplementary Table 2).

#### DNA isolation, verrucomicrobial 16S rDNA T-RFLP fingerprinting and quantitative real-time PCR assay

Soil DNA was extracted using the same conditions used for Model I. T-RFLP analysis was used to characterize the verrucomicrobial community structure in sugarcane rhizosphere soils from healthy and nutrient deficient symptom plants collected on the optimal and deficient soil fertility for sugarcane, respectively. T-RFLP fingerprinting of verrucomicrobial 16S rRNA genes, and qPCR analysis of verrucomicrobial and bacterial 16S rRNA genes were performed as described previously for Model I.

## Results

### Model I: slash-and-burn deforestation

**Fig. 1:**
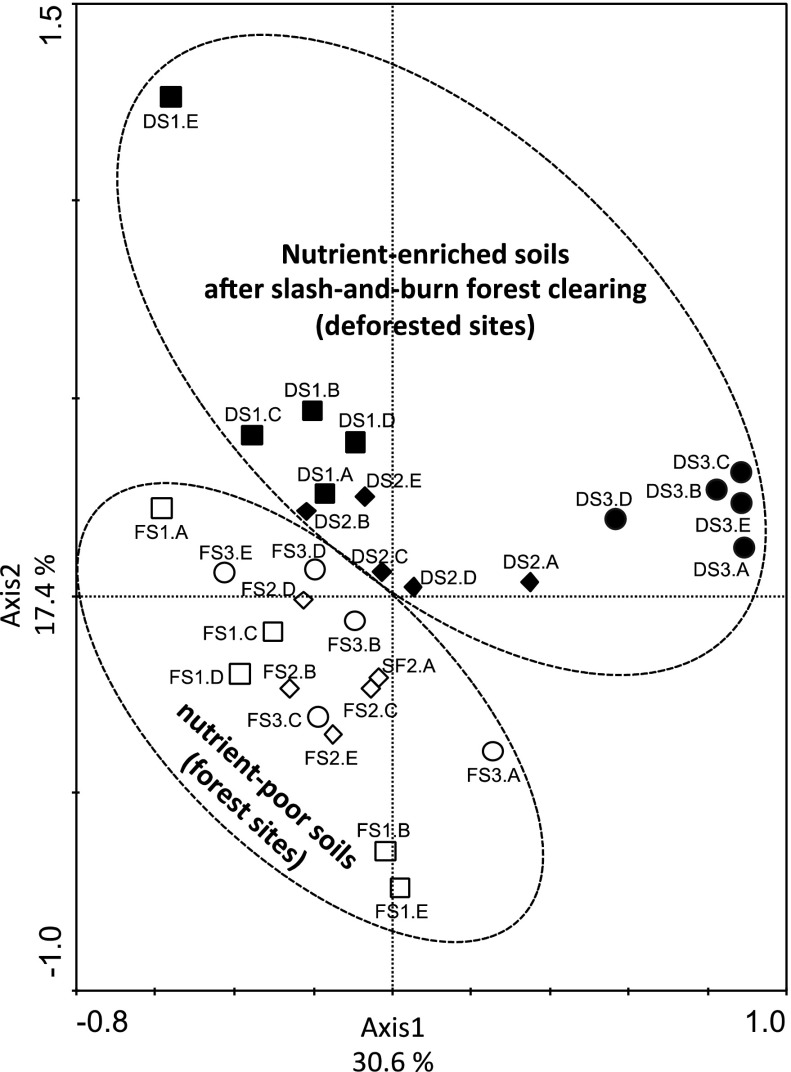
Principal component analysis plot based on the structure of soil verrucomicrobial communities as determined by T-RFLP analysis in nutrient-enriched soils after slash-and-burn forest clearing and natural nutrient-poor soils in an adjacent primary forest in three discontinuous areas after forest clearing and burning. *Symbols* refer to individual replicates (*A*, *B*, *C*, *D* and *E*) of the following sampling sites: *open squares* forest site located at Area 1 (FS1), *open diamonds* forest site located at Area 2 (FS2), *open circle* forest site located at Area 3 (FS3), *black squares* deforested site located at Area 1 (DS1), *black diamonds* deforested site located at Area 2 (DS2), *black circle* deforested site located at Area 3 (DS3)

PCA ordination based on verrucomicrobial T-RFLP data from restriction profiles generated by enzymes *Alu*I, *Msp*I and *Hha*I revealed distinct groups for nutrient-enriched soils after slash-and-burn deforestation and natural nutrient-poor soils under adjacent primary forest (Fig. 1). This grouping pattern was confirmed by a significant *R*-value (*R* = 0.819, *P* = 0.002) by analysis of similarity (ANOSIM) of the presence and absence of terminal restriction fragments in T-RFLP profiles. Soil samples from deforested sites were distinctly grouped in the ordination (*R* = 0.610, *P* = 0.003). However, verrucomicrobial community structure did not group according to the sampling sites in forest soils (*R* = 0.173, *P* = 0.003).

**Fig. 2:**
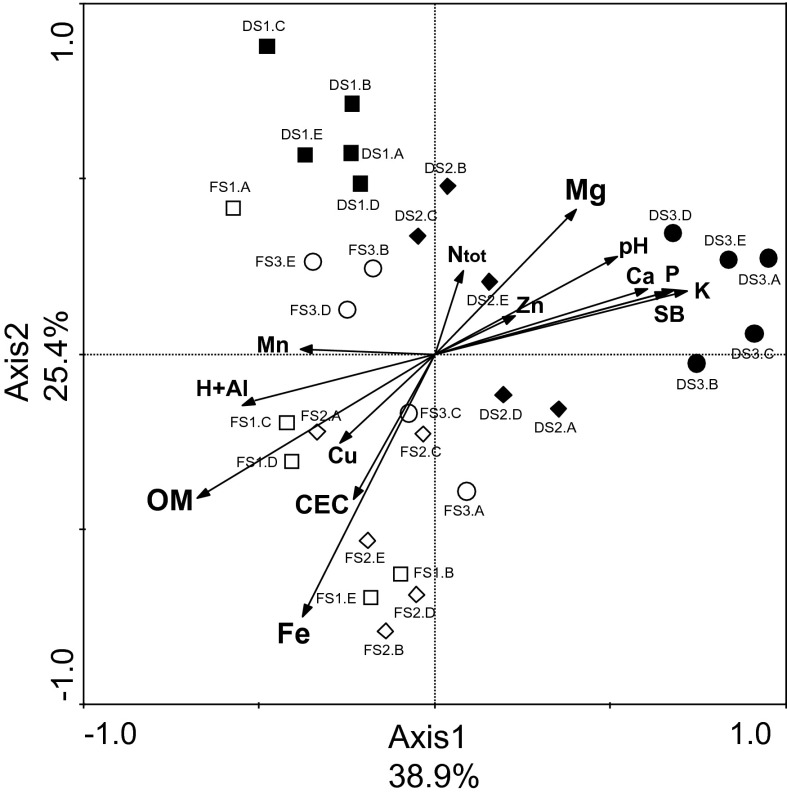
Constrained ordination diagram for sample plots (deforested and forest soil samples) in the first two redundancy analysis (RDA) axes based on the soil chemical characteristics of the different sampling sites and their relationship with the verrucomicrobial T-RFLP generated by restriction with enzymes *Alu*I, *Msp*I and *Hha*I. *Symbols* refer to individual replicates (*A*, *B*, *C*, *D* and *E*) of the following sampling sites: *open squares* forest site located at Area 1 (FS1), *open diamonds* forest site located at Area 2 (FS2), *open circle* forest site located at Area 3 (FS3), *black squares* deforested site located at Area 1 (DS1), *black diamonds* deforested site located at Area 2 (DS2), *black circle* deforested site located at Area 3 (DS3)

The relationship between soil chemical characteristics and verrucomicrobial community structures revealed by T-RFLP fingerprinting for nutrient-enriched soils after slash-and-burn deforestation (deforested soils) and natural nutrient-poor soils (forest soils) were calculated by redundancy analysis (RDA). A total of 64.3 % of all variation was explained by the first two RDA axes (Fig. 2). RDA results showed that community structures from deforested soils were related to soil chemical factors linked to soil fertility, such as total nitrogen (Ntot), phosphorus (P), potassium (K), calcium (Ca), magnesium (Mg), sum of bases (the sum of Ca, Mg and K), and soil pH. Verrucomicrobial community structures from forest soils were related to manganese (Mn), copper (Cu), iron (Fe) and organic matter (OM) content as well as cation exchange capacity (CEC) and potential acidity (H + Al) (Fig. 2).

Real-time quantitative PCR revealed significant differences (*P* < 0.05) in 16S rRNA gene copies between deforested soils and forest soils with regard to the relative and absolute proportions of the verrucomicrobial community in each of three replicates of sampling area (Table 1). *Verrucomicrobia* accounted, on average, for 4 and 2 % of the total bacterial signal in the soil samples from forest and deforested sites, respectively.

**Table 1:** Abundance of soil total bacteria and verrucomicrobial communities based on quantitative real-time PCR measurements in three discontinuous areas in the southeastern Brazilian Amazon in forested (FS) and deforested (DS) sites

	Area 1		Area 2		Area 3		Statistics
	FS	DS	FS	DS	FS	DS	FS versus DS
Absolute abundance (10^7^ 16S rDNA copies g soil)							
Total bacteria	14.2^a^a^b^ (±0.7)^c^	11.9a (±3.7)	13.3a (±2.1)	11.3a (±4.4)	18.0a (±2.0)	15.7a (±0.6)	ns^d^
*Verrucomicrobia*	0.56a (±0.01)	0.32b (±0.06)	0.61a (±0.07)	0.29b (±0.01)	0.76a (±0.01)	0.30b (±0.01)	**
Relative abundance (%)							
*Verrucomicrobia*	3.9a (±0.42)	2.7b (±0.62)	4.6a (±0.33)	2.6b (±0.22)	4.2a (±0.5)	1.9b (±0.66)	**

*FS* Soil from forest site, *DS* soil from deforested site. 1, 2 and 3 indicate three discontinuous sampling areas

^a^Average for each of five replicates soil

^b^Values with the same letters were not significantly different (*P* < 0.05)—within the same line—based on upon a Tukey’s HSD test followed by Bonferroni correction for multiple comparisons. Tukey’s test was performed contrasting FS versus DS within each area for each soil chemical factor across five soil cores for FS and five soil cores for DS

^c^Standard deviation of the average for each of five replicates soil

^d^Tukey’s HSD test followed by Bonferroni correction for multiple comparisons was performed considering FS versus DS regarding to all sampling sites across 15 soil cores for FS and 15 soil cores for DS. Significance levels: *ns* *P* > 0.05; ** *P* < 0.005

### Model II: management practices for sugarcane

**Fig. 3:**
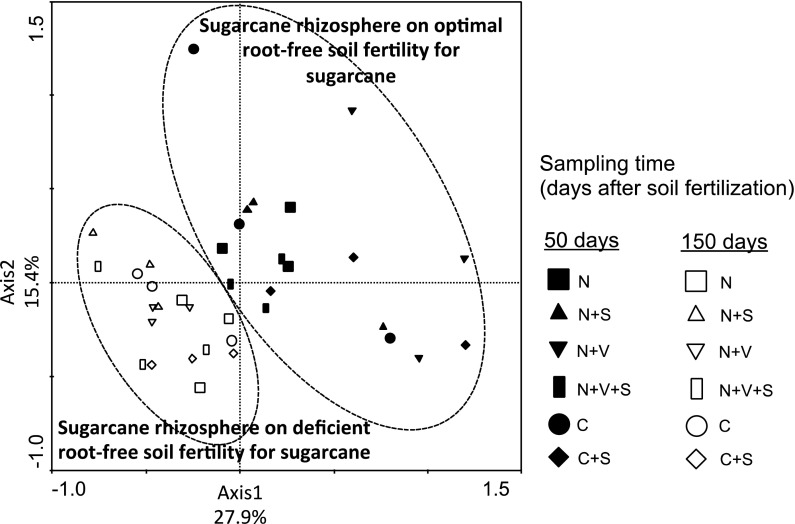
Principal component analysis-plot based on the structure of verrucomicrobial communities as determined by T-RFLP analysis in sugarcane rhizosphere soil collected from plants grown in a greenhouse mesocosm experiment and sampled on optimal and deficient root-free soil fertility for sugarcane

PCA ordination of the T-RFLP profiles generated by enzymes *Alu*I, *Msp*I and *Hha*I revealed distinct groups of soil *Verrucomicrobia* under optimal and deficient soil fertility for sugarcane cultivation (Fig. 3). Disparate grouping was confirmed by a significant *R*-value (*R* = 0.786, *P* = 0.002) verified by ANOSIM based on the presence and absence of terminal restriction fragments in T-RFLP profiles. Verrucomicrobial community structure did not group according to the experimental treatments (*R* = 0.244, *P* = 0.002).

**Fig. 4:**
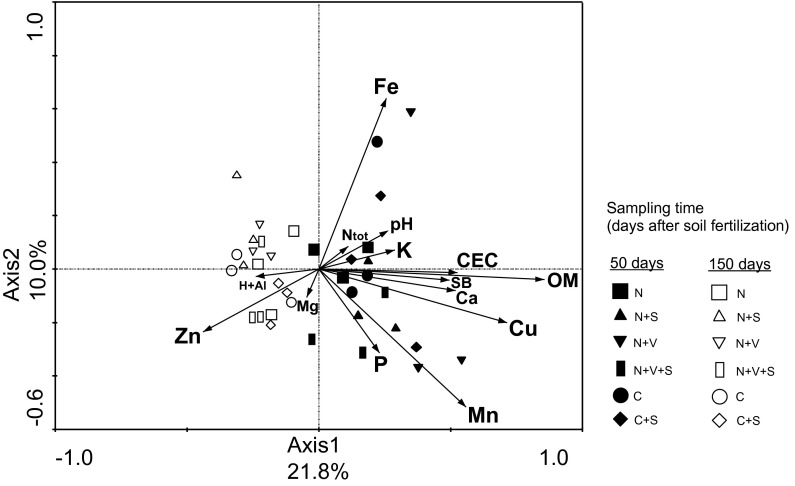
Constrained ordination diagram for sample plots (sugarcane rhizosphere soil samples collected on optimal and deficient soil fertility for sugarcane) in the first two redundancy analysis (RDA) axes based on the soil chemical characteristics of the different soil treatments and their relationship with the verrucomicrobial T-RFLP data from restriction profiles generated by enzymes *Alu*I, *Msp*I and *Hha*I

RDA showed relationships between soil chemical characteristics and verrucomicrobial community structures for sugarcane rhizosphere soil collected at optimal and deficient soil fertility for sugarcane. A total of 31.8 % of all variation was explained by the first two RDA axes (Fig. 4). Based on this multivariate analysis, the verrucomicrobial community structures from sugarcane rhizosphere receiving the optimal soil fertility for sugarcane were related to total N, P, K, Ca, Mn, Cu, Fe and OM contents, and soil pH and CEC (Fig. 4). In turn, verrucomicrobial community structures from sugarcane rhizosphere collected in nutrient-deficient soils were related to Zn and Mg contents as well as H + Al (Fig. 4).

ANOVA analyses were carried out on qPCR data targeting 16S rRNA gene abundances for total bacteria, as well as *Verrucomicrobia* (Table 2). These analyses did not show significant differences for total bacteria abundance between soil samples collected at the optimal and deficient soil fertility for sugarcane in any experimental treatments. However, the total number of 16S rRNA gene copies of rhizosphere *Verrucomicrobia* was significantly different between these two soil fertility conditions (Table 2). The proportion of the bacterial community that could be attributed to the phylum *Verrucomicrobia*, as based upon 16S rRNA gene copy detection, varied among soil samples from different experimental treatments, and it accounted, on average, for 2 and 5 % of the total bacterial signal, for soil samples collections taken from the optimal and deficient fertility soil, respectively (Table 2).

**Table 2:** Abundance of total bacteria and verrucomicrobial communities in soil based on quantitative real-time PCR measurements in sugarcane rhizosphere grown in a greenhouse mesocosm experiment and collected at optimal and deficient soil fertility for sugarcane

	Sugarcane rhizosphere on optimal soil fertility for sugarcane						Sugarcane rhizosphere on deficient soil fertility for sugarcane					
	N	N + S	N + V	N + V + S	C	C + S	N	N + S	N + V	N + V + S	C	C + S
Absolute abundance (10^7^ 16S rDNA copies g soil)												
Total bacteria	12.2^a^a^b^ (±0.6)^c^	12.9a (±0.7)	13.5a (±0.6)	14.1a (±0.6)	12.3a (±0.7)	12.7a (±0.5)	13.6a (±0.7)	13.8a (±0.6)	14.7a (±0.8)	14.9a (±0.4)	12.4a (±0.6)	12.9a (±0.8)
*Verrucomicrobia*	0.28a (±0.02)	0.33a (±0.03)	0.27a (±0.01)	0.29a (±0.04)	0.23a (±0.03)	0.25a (±0.05)	0.65b (±0.08)	0.74b (±0.07)	0.66b (±0.08)	0.74b (±0.09)	0.57b (±0.07)	0.63b (±0.06)
Relative abundance (%)												
*Verrucomicrobia*	2.3a (±0.4)	2.6 (±0.3)	2.0 (±0.5)	2.1 (±0.4)	1.9 (±0.5)	2.0 (±0.6)	4.8b (±0.7)	5.4 (±0.3)	4.5 (±0.4)	5.0 (±0.5)	4.6 (±0.8)	4.9 (±0.7)

Experimental treatments: N, nitrogen fertilizer; N + S, N fertilizer and straw blanket; N + V, N and vinasse as fertilizers; N + V + S, N and V as fertilizers and straw blanket; C, excluding any N, V fertilizer and straw blanket (control); and C + S, excluding any N and V fertilizer and including straw blanket

^a^Average for each of six replicates sugarcane rhizosphere soil

^b^Values with the same letters were not significantly different (*P* < 0.05)—within the same line—based on upon a Tukey’s HSD test followed by Bonferroni correction for multiple comparisons. Tukey’s HSD test was performed contrasting optimal versus deficient soil fertility conditions for surgarcane within each experimental treatment

^c^Standard deviation of the average for each of six replicates soil

## Discussion

Soils of the Amazon ecosystem are extremely low in all nutrients (Aubert and Tavernier 1972), characterized by low natural fertility, high exchangeable aluminium saturation, aluminium toxicity, K deficiency, high P fixation and low CEC (Cochrane and Sanchez 1982; Mendonça-Santos et al. 2006). Cultivation of acid soils in the Amazon is preceded by cutting and removing the economically important trees and burning the remaining aerial biomass (Fujisaka et al. 1996). Certini (2005) revised the effects of fire on properties of forest soils and reported that slash-and-burn deforestation contributes to the availability of micro- and macronutrients in the soil by releasing nutrients from OM. The resulting changes in soil nutrient availability in turn affects soil C and N dynamics by accelerating OM decomposition (Certini 2005). Navarrete et al. (2015b) reported short-term increase in soil fertility after slash-and-burn deforestation in Amazonia based on chemical analysis of the soil fertility status for the same soil samples used in the present study. The authors showed that slash-and-burn forest clearing in Amazonia decreased soil organic matter (OM) content and factors linked to soil acidity, and raised soil pH, base saturation, and the concentration of exchangeable bases. Nye and Greenland (1960) proposed the ‘nutrient-rich ash’ hypothesis to explain the observed increase in soil nutrient availability after slash-and-burn deforestation. The increased availability of nutrients from ash incorporation and soil OM combustion lead to increased soil fertility, with effects on soil bacterial communities composition and their potential functions (Navarrete et al. 2015b).

The distribution of the *Verrucomicrobia* phylum in soils (including plant rhizosphere soils) is variable and apparently extremely sensitive to changes in the environment (Kielak et al. 2008; Bruce et al. 2010; Pan et al. 2014; Navarrete et al. 2015a, b). Buckley and Schmidt (2001) showed that the distribution of rRNA from *Verrucomicrobia* in soil was affected by environmental characteristics that change in relation to time, soil history, and soil depth, and revealed that a statistically significant amount of the variation in verrucomicrobial 16S rRNA genes abundance can be explained by changes in soil moisture content together with other soil factors. In the present study, we showed that the verrucomicrobial community structure and abundance were affected by soil chemical factors linked to soil fertility, such as total N, P, K and sum of bases, i.e., the sum of calcium, magnesium and K contents.

Wertz et al. (2012) considered that *Verrucomicrobia* may exert a great impact with regard to nitrogen availability in certain ecosystems, including oligotrophic environments. This consideration was based on genomic data from TAV2, which is a member of the *Opitutaceae* from the gut of the termite *Reticulitermes flavipes*, and genomic data from the as-yet-unpublished *Verrucomicrobium* strain DG1235. As termite diets have a low N content, *Verrucomicrobia* may contribute the N pool within the gut ecosystem. Isanapong et al. (2013) showed some *Verrucomicrobia* seem to be well adapted to environments with low nitrogen availability by carrying genes for biosynthesis of amino acids and transcribing of those genes in situ. In our soils, higher verrucomicrobial community abundance was related to low N availability.

Huang et al. (2012) reported a decrease in abundance of *Verrucomicrobia* after increases in available nitrogen, phosphorus and potassium, and soil OM resulting from cotton straw application in soil. Pitombo et al. (2015) also found a negative effect of straw application in sugarcane on abundance of members of *Verrucomicrobia*. A negative effect of K content on abundance of *Verrucomicrobia* was found in grassland soil with yearly addition of inorganic fertilizers in a 54-year experiment (Pan et al. 2014) and sugarcane-cultivated soil with addition of vinasse in a short-term experiment (Navarrete et al. 2015a). Lima et al. (2014) showed that K also negatively affected *Verrucomicrobia* in Amazonian Dark Earth, one of the most fertile soils in the world. In addition, Mendes et al. (2015) reported lower abundance of *Verrucomicrobia* in deforested, agricultural and pasture soils than primary forest soils in Brazilian Amazonia, where the K content was higher than undisturbed soils. Our findings on verrucomicrobial community structure and abundance corroborate the previous findings that higher verrucomicrobial community abundance is associated with soils containing lower P and K contents.

Members of the phylum *Verrucomicrobia* have been shown to be present in varying plant-soil ecosystems (Chow et al. 2002; DeAngelis et al. 2009; Kielak et al. 2008; Nunes da Rocha et al. 2011, 2013; Sanguin et al. 2006; Zul et al. 2007). However, to date, there is no information about the verrucomicrobial community inhabiting the sugarcane rhizosphere soil. Nunes da Rocha et al. (2013) found specialized groups within several subdivisions of *Verrucomicrobia* that are leek rhizosphere competent. According to the authors, the lifestyles of these specialized groups of *Verrucomicrobia* with the plant may suggest that these bacteria either interact with the plants themselves or with other microbial communities associated with plants. Our results extend this current understanding of verrucomicrobial communities that associate with plants and suggest that these bacteria also interact with the soil chemical factors of the root-free soil surrounding of sugarcane plant roots, especially those related to soil fertility. The verrucomicrobial community structures from sugarcane rhizosphere given the optimal soil fertility for sugarcane were related to total N, P, K, Ca, Mn, Cu, Fe and OM contents. With regard to the relationship between *Verrucomicrobia* and soil OM content, the generally oligotrophic phylum *Verrucomicrobia* may be highly dependent on C availability due to a slow-growing life strategy (Bergmann et al. 2011; He et al. 2012). In this sense, although the effects of sugarcane crop residue on soil biological properties are expected to be more pronounced over long time periods (Graham et al. 2002), our results showed a tendency for increased abundance of *Verrucomicrobia* in soil with straw blanket coverage.

In our study, forest soils and sugarcane-cultivated soils on deficient fertility are considered to be oligotrophic since they contain low levels of nutrients and deforested soils and sugarcane-cultivated soils on optimal fertility as copiotrophic environments due to increase in nutrient availability after slash-and-burn forest clearing and fertilization, which increases nutrient availability (Carbonetto et al. 2014; Navarrete et al. 2015b). Under this assumption, bacteria in forest soils and sugarcane-cultivated soils on deficient fertility are expected to be *k*-selected and to present low growth rates and very efficient nutrient uptake systems with higher substrate affinities. In contrast, bacteria in deforested soils and sugarcane-cultivated soils on optimal fertility are expected to be *r*-selected and to have higher rates of activity per biomass unit, higher turnover rates and faster growth rates. Our results showed a trend toward these statements since a reduction in the abundance of *Verrucomicrobia,* a bacterial taxon with oligotrophic characteristics (Carbonetto et al. 2014; Navarrete et al. 2015b), was detected in deforested soils and sugarcane-cultivated soils on optimal fertility. Moreover, this is in agreement with recent findings that confirm a correlation between *Verrucomicrobia* abundance patterns and conditions of limited nutrient availability in tropical soil (Navarrete et al. 2015b). Culture-independent analysis of soil microbial communities revealed *Verrucomicrobia* as a bacterial phylum adapted to low substrate concentrations in soil (Noll et al. 2005). High-throughput sequencing of the same soil DNA samples used in this study revealed a high abundance of *Verrucomicrobia* at the phylum and class (*Spartobacteria*) levels in Amazon forest soils (Navarrete et al. 2015b), where the fertility is naturally low and maintained through litter nutrient cycling under high moisture condition (Fearnside 2005).

Based on our case studies (Models I and II), a clear link has been established between soil verrucomicrobial community structure and abundance and soil fertility status. Verrucomicrobial communities in the studied soils are consistent with an oligotrophic lifestyle, revealing decreased abundance and disparate community structure pattern on nutrient-enriched soils in comparison to nutrient-deficient soils. In conclusion, the community structure and abundance represent important ecological aspects in soil verrucomicrobial communities for tracking the changes in chemical factors linked to soil fertility under slash-and-burn deforestation and management practices for sugarcane.

## Electronic supplementary material

Supplementary material 1 (DOCX 21 kb)
