# Supplementary material for: Verrucomicrobial community structure and abundance as indicators for changes in chemical factors linked to soil fertility
Source: Antonie Van Leeuwenhoek. 2015 Jul 17;108(3):741–52. doi: 10.1007/s10482-015-0530-3 (PMC4525199; doi:10.1007/s10482-015-0530-3)
Supplement: Supplementary file 1 — Supplementary material 1 (DOCX 21 kb) [file 10482_2015_530_MOESM1_ESM.docx]

**Supplementary Table 1**. Soil chemical factors of the 0- to 20-cm topsoil layer at forest and deforested sites from three discontinuous areas in the Brazilian Amazon

| **Soil factors** | **Area 1** | |  | **Area 2** | |  | **Area 3** | | **Statistics** |
| --- | --- | --- | --- | --- | --- | --- | --- | --- | --- |
|  | **Forest**  **site** | **Deforested site** |  | **Forest**  **site** | **Deforested site** |  | **Forest**  **site** | **Deforested site** | **FS *vs.* DS** |
|  |  |  |  |  |  |  |  |  |  |
| pH | 4.76^*^a† ± 0.3‡ | 5.02a ± 0.1 |  | 4.9a ± 0.4 | 5.32a ± 0.1 |  | 4.6a ± 0.2 | 5.26a ± 0.1 | ***^\|\|^ |
| OM | 54.8a ± 5.1 | 39.6b ± 3.6 |  | 58.6a ± 21.6 | 39.8b ± 4.1 |  | 31.8a ± 1.8 | 15.8b ± 8.7 | * |
| Ntot | 1.30a ± 0.03 | 1.70b ± 0.04 |  | 1.30a ± 0.02 | 1.73b ± 0.04 |  | 1.32a ± 0.02 | 1.75b ± 0.05 | ** |
| P | 1.0a ± 0 | 1.4a ± 0.9 |  | 1.0a ± 0 | 1.2a ± 0.4 |  | 4.8a ± 0.8 | 5.8a ± 1.6 | ns |
| K | 0.9a ± 0.1 | 0.46b ± 0.2 |  | 0.7a ± 0.1 | 0.44a ± 0.3 |  | 0.4a ± 0.05 | 0.34a ± 0.05 | ** |
| Ca | 2.0a ± 0.7 | 3.8a ± 1.6 |  | 1.4b ± 0.5 | 12.2a ± 6.2 |  | 2.4b ± 2.6 | 9.6a ± 6.3 | *** |
| Mg | 2.0a ± 0.7 | 2.0a ± 1.0 |  | 1.4b ± 0.5 | 4.6a ± 2.2 |  | 1.0b ± 0 | 8.8a ± 6.8 | ** |
| H+Al | 89.4a ± 8.6 | 70.0b ± 5.6 |  | 102.4a ± 40.9 | 57.6b ± 8.5 |  | 61.4a ± 4.7 | 40.4b ± 6.9 | *** |
| SB | 4.8a ± 1.4 | 6.32a ± 2.4 |  | 3.48b ± 0.9 | 16.9a ± 8.4 |  | 3.74b ± 2.6 | 18.8a ± 13.2 | ** |
| CEC | 94.0a ± 9.6 | 76.2b ± 7.4 |  | 106.2a ± 40.6 | 74.6a ± 9.9 |  | 65.1a ± 5.6 | 59.2a ± 10.4 | * |
| Cu | 0.3a ± 0.05 | 0.2b ± 0.04 |  | 0.3a ± 0.04 | 0.3a ± 0.08 |  | 0.2a ± 0.07 | 0.2a ± 0.04 | ns |
| Fe | 228.6a ± 62.4 | 85.6b ± 17.0 |  | 227.4a ± 96.5 | 77.8b ± 10.0 |  | 120.0a ± 32.3 | 76.2a ± 38.4 | *** |
| Mn | 4.0a ± 3.0 | 2.0a ± 0.6 |  | 1.7a ± 0.9 | 3.3a ± 1.4 |  | 1.6a ± 0.4 | 0.6b ± 0.1 | ns |
| Zn | 0.3a ± 0.1 | 0.3a ± 0.05 |  | 0.2a ± 0.07 | 0.2a ± 0.05 |  | 0.3a ± 0.08 | 0.3a ± 0.2 | ns |

Adapted from Navarrete et al. (2015)

FS, soil from forest site; DS, soil from deforested site. 1, 2 and 3 indicate different sampling areas

Ca, Mg, K, Al, potential acidity (H+Al), sum of base (SB) are expressed in mmol_c_.kg^-1^; OM is expressed in g.kg^-1^; total soil nitrogen (Ntotl) is expressed in mg.g^-1^; P is expressed in mg.kg^-1^; Fe, Mn, Zn, K and cation exchange capacity in pH 7 (CEC) are expressed in mg.dm^-3^. P-K – Mehlich 1 extractor. Ca and Mg – KCl 1N. H+Al – SMP extractor. OM – organic matter. SB – sum of bases

* The values are averages based on quintuplicate sampling points in each site

† Values with the same letters were not significantly different (*P*<0.05) based on upon a Tukey’s HSD test followed by Bonferroni correction for multiple comparisons. Tukey’s test was performed contrasting FS *vs*. DS within each area for each soil chemical factor across five soil cores for FS and five soil cores for DS.

‡ Standard deviation of the average for each of five replicates soil

^||^ Tukey’s HSD test followed by Bonferroni correction for multiple comparisons was performed considering FS *vs.* DS regarding to all sampling sites across 15 soil cores for FS and 15 soil cores for DS. Significance levels: ns: *P*>0.05; ***P*<0.005, *** *P*<0.0005

**Supplementary Table 2**. Soil chemical factors of the 0- to 10-cm topsoil layer of sugarcane-cultivated soils at optimal and deficient soil nutrients in a greenhouse mesocosm experiment.

| Chemical factors |  | 50 days after planting and soil fertilization  (optimal soil fertility for sugarcane) | | | | | |  | 150 days after planting and soil fertilization  (deficient soil fertility for sugarcane) | | | | | |
| --- | --- | --- | --- | --- | --- | --- | --- | --- | --- | --- | --- | --- | --- | --- |
|  |  | N | N+S | N+V | N+V+S | C | C+S |  | N | N+S | N+V | N+V+S | C | C+S |
| pH |  | 5.2*a† ± 0.05‡ | 5.1a ±0.1 | 5.5b ± 0.5 | 5.4a ± 0.1 | 5.3a ± 0.1 | 5.2a ± 0.1 |  | 5.2a ± 0.05 | 5.2a ± 0.05 | 5.9a ± 0.1 | 5.6a ± 0.1 | 5.4a ± 0.1 | 5.2a ± 0.1  26.7a ± 0.8 |
| OM |  | 32.0a ± 0.8 | 33.0a ± 1.4 | 36.5a ± 0.6 | 36.5a ± 2.08 | 25.2a ± 1.28 | 26.1a ± 1.7 |  | 28.1a ± 0.5 | 28.9a ± 0.8 | 27.7a ± 0.9 | 28.2a ± 1.5 | 26.0a ± 1.5 |  |
| Ntot |  | 1.9a ± 0.02 | 1.9a ± 0.01 | 1.9b ± 0.01 | 1.9 ± 0.02 | 1.9a ± 0 | 1.9a ± 0.01 |  | 1.8b ± 0.01 | 1.8b ± 0.02 | 2.0a ± 0.02 | 1.9a ± 0.01 | 1.8b ± 0.02 | 1.8b ± 0.02 |
| P |  | 97.5a ± 18.9 | 94.2a ± 30.0 | 63.5a ± 21.3 | 113.2a ± 61.6 | 75.7a ± 15.0 | 90.2a ± 39.7 |  | 77.2a ± 31.4 | 41.7b ± 4.7 | 55.0a ± 8.7 | 69.0b ± 17.5 | 50.2a ± 17.0 | 55.5a ± 17.8 |
| K |  | 3.6a ± 0.6 | 3.7a ± 0.7 | 15.6a ± 3.1 | 11.3a ± 1.7 | 3.8a ± 0.4 | 3.0a ± 0.6 |  | 2.9a ± 0.8 | 2.2a ± 0.8 | 13.0a ± 3.5 | 5.8a ± 1.2 | 2.5a ± 0.3 | 2.5a ± 0.4 |
| Ca |  | 55.2a a± 2.2 | 54.7a ± 2.1 | 55.7a ± 1.7 | 54.5a ± 3.0 | 59.5a ± 3.1 | 57.2a ± 1.5 |  | 52.5a ± 8.9 | 49.5b ± 2.1 | 51.2a ± 4.0 | 49.5b ± 1.9 | 56.7a ± 4.3 | 52.5b ± 3.3 |
| Mg |  | 16.0a a± 1.4 | 15.2a ± 1.2 | 17.5a ± 1.0 | 17.5b ± 0.6 | 15.7a ± 0.9 | 16.0a ± 0.8 |  | 16.0a ± 4.1 | 15.0a ± 0 | 17.2a ± 1.5 | 20.0a ± 3.4 | 17.2a ± 2.2 | 16.2a ± 0.9 |
| H+Al |  | 45.7a ± 2.5 | 40.2a ± 4.5 | 34.2a ± 2.9 | 40.5a ± 7.9 | 34.2a ± 2.9 | 37.0a ± 3.8 |  | 34.0a ± 0 | 34.2a ± 2.9 | 23.5a ± 1.7 | 28.7a ± 1.5 | 28.0a ± 0 | 31.7a ± 1.5 |
| SB |  | 81.1a ± 4.3 | 78.8a ± 3.6 | 79.9a ± 3.0 | 72.2a ± 4.1 | 98.9a ± 10.7 | 92.3a ± 9.5 |  | 76.2a ± 5.5 | 70.8a ± 3.4 | 71.5b ± 2.1 | 66.4a ± 1.7 | 84.3a ± 11.0 | 75.5b ± 3.9 |
| CEC |  | 122.7a ± 4.6 | 113.5a ± 9.3 | 120.3a ± 4.2 | 120.7a ± 5.7 | 115.2a ± 6.5 | 112.8a ± 5.8 |  | 105.6b ± 14.0 | 100.7a ± 3.3 | 107.9a ± 11.7 | 103.9b ± 5.0 | 103.9a ± 5.5 | 102.4b ± 2.1 |
| Cu |  | 1.0a ± 0.05 | 1.0a ± 0.05 | 0.9a ± 0.1 | 1.9a ± 0.05 | 1.0a ± 0.05 | 1.0a ± 0.05 |  | 0.5b ± 0.1 | 0.6b ± 0.2 | 0.6b ± 0.1 | 0.6b ± 0.1 | 0.6b ± 0.2 | 0.7b ± 0.2 |
| Fe |  | 36.0a ± 3.2 | 38.0a ± 3.4 | 46.2a ± 30.5 | 38.7a ± 4.3 | 33.5a ± 3.7 | 33.7a ± 2.6 |  | 38.0a ± 3.4 | 37.0a ± 3.0 | 37.5a ± 0.6 | 37.2a ± 1.7 | 31.5a ± 0.6 | 36.2a ± 3.0 |
| Mn |  | 11.4a ± 0.8 | 13.0a ± 2.7 | 13.6a ± 7.2 | 16.3a ± 1.3 | 10.9a ± 1.4 | 11.1a ± 1.2 |  | 7.9b ± 0.3 | 7.3b ± 1.0 | 8.5a ± 1.0 | 8.5b ± 0.4 | 6.7b ± 0.3 | 7.7b ± 0.6 |
| Zn |  | 2.8a ± 1.3 | 2.4a ± 0.7 | 2.2b ± 1.3 | 2.0b ± 0.7 | 1.8b ± 0.2 | 1.8b ± 0.2 |  | 3.9a ± 0.8 | 4.0a ± 3.4 | 13.1a ± 6.9 | 5.1a ± 1.3 | 3.6a ± 1.0 | 3.1a ± 1.3 |

Adapted from Navarrete et al. (2015a)

N = nitrogen as fertilizer; V = *vinasse* as fertilizer; S = straw blanket; C = control - without any N and V fertilizer

Ca, Mg, K, Al, potential acidity (H+Al), sum of base (SB) are expressed in mmol_c_.kg^-1^; organic matter (OM) is expressed in g.kg^-1^; total soil nitrogen (Ntot) is expressed in mg.g^-1^; P is expressed in mg.kg^-1^; Fe, Mn, Zn, K and cation exchange capacity in pH 7 (CEC) are expressed in mg.dm^-3^. P-K – Mehlich 1 extractor. Ca and Mg – KCl 1N. H+Al – SMP extractor.

*Average for each of three replicates of soil

Tukey’s HSD test was performed separately for each experimental treatment

†Values with the same letters were not significantly different (*P*<0.05) based on upon a Tukey’s HSD test. Tukey’s HSD test was performed considering samples collected at optimal *vs*. deficient soil fertility condition for sugarcane within each experimental treatment.

‡Standard deviation of the average for each of three replicates of soil
